# Evaluation of *Withania somnifera* based supplement for immunomodulatory and antiviral properties against viral infection

**DOI:** 10.1016/j.jaim.2024.100955

**Published:** 2024-10-09

**Authors:** Dileep Kumar Verma, Abdul Hasan, Manickavasagam Rengaraju, Shree Devi, Geetika Sharma, Vimal Narayanan, Sathiyarajeswaran Parameswaran, Thirumal Kumar D, Kanakavalli Kadarkarai, Sujatha Sunil

**Affiliations:** a Vector-Borne Disease Group, International Center for Genetic Engineering and Biotechnology (ICGEB), New Delhi, India; b Siddha Clinical Research Unit, Govt. Sri Jayachamarajendra Institute of Indian Medicine Campus, Bengaluru, Karnataka, India; c Siddha Central Research Institute, Chennai, India; d Santhigiri Research Foundation, Santhigiri Ayurveda and Siddha Hospital, Bengaluru, Karnataka, India; e Faculty of Allied Health Sciences, Meenakshi Academy of Higher Education and Research (MAHER), Chennai, India; f Central Council for Research in Siddha, Chennai, India

**Keywords:** SARS-CoV-2, Siddha, Nutraceutical, MAM granules, Antioxidant, Inflammation, Antiviral, *Withania somnifera* (Amukkara), *Piper nigrum* (Milagu), *Curcuma longa* (Manjal)

## Abstract

**Background:**

Viral mediated diseases are continuously posing potent threat to human health. Nutraceuticals are being employed as novel therapeutics during viral outbreaks. MAM granules consist of *Curcuma longa*, *Withania somnifera*, and *Piper nigrum*, is one such patented Siddha nutraceutical supplement that has been proposed to be a therapeutic agent against viral diseases.

**Objective:**

We characterised MAM for their phytochemical and physicochemical properties and evaluated its cytotoxicity via *in vivo* acute toxicity studies using Wistar rats and by cell-based MTT assays.

**Materials and Methods:**

The antiviral properties of the aqueous extract of MAM were investigated against SARS-CoV-2 and chikungunya virus (CHIKV). Further, using ABTS radical scavenging, SOD enzymatic assays and measurement of intracellular ROS, we investigated the antioxidant potential of MAM extract and its ingredients in RAW264.7 cells. Additionally, production of inflammatory mediators was evaluated via NO release, PGE2 production and release of pro-inflammatory cytokines (IL-1*β* and TNF*α*).

**Results:**

The MAM granules and aqueous extracts demonstrated no significant toxicity and demonstrated potent antiviral activity during co-incubation assay with SARS-CoV-2 and CHIKV. Moreover, we observed potent antioxidant and anti-inflammatory activity of MAM extract in a dose dependent manner. Further investigations on the individual ingredients with respect to their antioxidant and anti-inflammatory activities showed that all ingredients contributed synergistically and *Withania somnifera* showed most potent anti-oxidant activity.

**Conclusion:**

The overall in vitro, and *in vivo* analysis demonstrated that MAM granules were non-toxic and possessed potent antiviral activity. Additionally, observed significant anti-oxidant and anti-inflammatory properties of MAM suggested the modulation of innate immune response in the host validating its use as an effective nutraceutical during viral outbreaks.

## 1 Introduction

Viral outbreaks impose major public health threats to human population and result in a heavy disease burden [1]. COVID-19, a respiratory viral disease outbreak caused by SARS-CoV-2 has significantly affected the global population [2,3]. Some of the viral outbreaks are seasonal, occurring simultaneously in the population complicating treatment and disease management. For instance, viral infections such as chikungunya, dengue affect population since the onset of monsoon owing to rise in vector population and present with overlapping symptoms and with the emergence of the COVID-19 pandemic, management of these diseases has become further confounding. In such a scenario, novel strategies such as usage of medicinal supplements and diet management may aid in disease management [4].

In recent years, scientific interests are growing in nutraceuticals with the concept of “Food is medicine; Medicine is Food”. Nutraceuticals are involved in the maintenance of well-being, modulation of the immune system, thus enhancing health. Nutraceuticals interact with the components of the immune system and improve the immune response to viral pathogens. Siddha is a traditional indigenous medical system widely recognized as an effective strategy for the prevention and treatment of a variety of diseases. Moreover, several studies had demonstrated the use of the Siddha medical system for the effective treatment of viral diseases [5,6]. MAM Granules is a provisionally patented Siddha nutraceutical supplement, made up of three ingredients viz. *Curcuma* *longa*, *Withania somnifera*, and *Piper nigrum* and have been evaluated for COVID-19 [7]. Moreover, the ingredients of MAM have been previously explored for their activity as ROS inhibitor, anti-inflammatory activity and antiviral activities. For example, *Curcuma longa,* [[8], [9], [10]] *Withania somnifera* [[11], [12], [13]], and *Piper nigrum* [[14], [15], [16]] have been reported to effectively reduce the increase in intracellular ROS and demonstrate anti-inflammatory activity. Similarly, the antiviral activity of *Withania somnifera* [17,18]*, Curcuma longa* [19] *and Piper nigrum* [20,21] for different viruses have been reported. Here, we explored their activity as a formulation i. e. MAM granules.

At the cellular level, one of the first consequences of virus infection is the induction of oxidative stress in the cell they infect and is characterized by increase in generation of free radicals and reactive oxygen/nitrogen species (ROS/RNS) [22]. The excessive production of these molecules in the cell further damages the cellular components [23]. This kick starts the cellular homeostasis machinery which employs different antioxidants to suppress the production of the ROS and amongst these, Superoxide Dismutase (SOD) works as first line defence mechanism [24,25]. The sudden spike in ROS and free radicals then activate inflammatory pathways and initiate cellular inflammatory responses. These responses are mediated by two key cellular factors i. e. Nitric Oxide (NO) and PGE2 which activates previously silent signalling pathways leading to translocation of transcriptional factors in the nucleus required for transcription of cellular genes to produce interferons and pro-inflammatory cytokines [26,27].

Use of traditional medicines for the treatment of infectious studies has proven to be an effective alternative to modern medicines [28,29]. These medicines are well reported to strengthen the immune system, thereby helping both an infected individual to fight the pathogen as well as aid in building the immunity of a healthy individual to fight an impending pathogen especially during viral outbreaks in a community [30]. With respect to the latter option, in recent times, impetus has been well thrusted on the concept of nutraceuticals as a preventive measure to combat infectious diseases. One such nutraceutical, MAM, has been developed using three ingredients viz. *Curcuma longa, Withania somnifera and Piper nigrum* and have been evaluated for its usage as effective supplement against viral diseases.

In the present study, we first evaluated the acute toxicity of the MAM granules in Wistar rats. Using an aqueous extract, we further evaluated its cytotoxicity using *in-vitro* cell-based assays in Vero-E6 and RAW264.7 cells. Using the maximum non-toxic dose (MNTD), we investigated the antiviral activity of aqueous extract of MAM using different types of antiviral assays on two RNA viruses i. e. SARS-CoV-2 and chikungunya virus (CHIKV). It was observed that co-incubating the MAM aqueous extract with both of these viruses significantly reduced viral titers. Next, we systematically evaluated the aqueous extract of this nutraceutical and its ingredients for their antioxidant and anti-inflammatory properties in RAW264.7 cells. We observed the aqueous extract of MAM exhibited strong anti-oxidant potential and was able to suppress inflammatory mediators suggesting potent anti-inflammatory capacity. The three ingredients of MAM extract i. e. *Curcuma longa, Withania somnifera* and *Piper nigrum* also showed significant anti-oxidant and anti-inflammatory capacity in a dose dependent manner without imposing significant cytotoxicity.

## 2 Material and methods

### 2.1 Rationale for the formulation and its Siddha Text references

As per the Siddha Literature, all 3 ingredients have anti-inflammatory and antioxidant properties. They are time-tested medicinal drugs and the therapeutic benefits of these ingredients individually indicated for the management of Suram (fever) which is mentioned as Suram in Siddha Text “Agathiyar Gunavagadam”. Based on Taste concept of Siddha, they are also potent drugs to treat Surams – Vida Suram/Suram/Seetha Suram) (Viral Fevers). So Based on Siddha Basic concepts and Therapeutic references, these 3 ingredients were selected and made into the formulation in the proper ratio. (MAM – 1:4:1).

### 2.2 Preparation of MAM granules/aqueous extract

The MAM granules were prepared based on an analysis of the properties of the individual components as per the existing Siddha text literature - Gunapadam Mooligai. *Curcuma* l*onga* (Manjal), *Withania somnifera* (Amukkara), and *Piper* n*igrum* (Milagu) were mixed in a ratio of 1:4:1 in this preparation. The root tuber of Aswagandha, Curcuma and dried fruits of Pepper were used to prepare the MAM Granules formulations. The raw drugs were purchased from the raw drug store. For the in-vitro studies, 100 g of polyherbal formulation was taken in 800 ml water and a decoction was prepared as per Gunapadam text literature. Decoction was filtered through muslin cloth to obtain 200 ml of aqueous extract, concentrated under vacuum using rotatory evaporator and lyophilized to remove the water content. Then, an aqueous solution was prepared of concentration 83.88 mg/ml in deionized water followed by vortex and kept for 30 min at 40 °C. Subsequently, solution was centrifuged at 3000 rpm for 10 min at RT and supernatant was collected, filtered and used for experiments.

### 2.3 Standardization and characterization of MAM extract

The raw drugs used in the preparation of MAM were procured from the local market of Chennai, Tamil Nadu, India. They were properly processed after authentication by Department of Pharmacognosy, Siddha Central Research Institute (SCRI), Arumbakkam, Chennai, Tamil Nadu, India. Certificate of Authentication No. 214.06021001 dated January 06, 2021. A voucher specimen of the same was deposited in the museum of Department of Pharmacognosy, SCRI, Chennai.

Organoleptic parameters such as color, odor, taste, shape, and size were analysed and recorded. Powder microscopy of shade-dried powder was carried out using Nikon ECLIPSE E 200 trinocular microscope attached to Zeiss ERc5s digital camera under bright field light [17]. Physico-chemical characteristics of MAM were analysed by quantitative analysis for total ash, water -soluble ash, acid-insoluble ash, water-soluble extractives, alcohol-soluble extractives, loss on drying, and pH (10% aqueous solution) as per standard techniques [18]. The preliminary phytochemical screening of MAM Granules was screened for the identification of phytochemical constituents by HPTLC Methods [19].

Inductively Coupled Plasma Optical Emission Spectroscopy (ICP-OES) is one of the most common techniques for elemental analysis and useful for standardization as well as to develop an analytical profile.

### 2.4 Animal toxicity experiments

An acute oral toxicity studies of MAM granules were performed on Wister rats (*Rattus norvegicus*) using the classical acute toxicity protocol. The procurement of animals was from Gentox Bio Services Pvt. Ltd., Hyderabad, with the following registration details - CPCSEA Registration No.: 1242/PO/RcBiBt/S/08/CPCSEA. The animals were acclimatized for a period of 9, 11, 13 and 15 days for Set I, Set, Set III and Set IV respectively in the experimental animal room before start of treatment. The animals were observed once daily for any abnormalities. All rats were maintained on a 12-h light/dark cycle and located at room temperature of approximately 25 ± 5 °C with constant humidity.

Four groups of female mice (nulliparous and non-pregnant) of age group 11–13 weeks and body weight ranging from 160.91 g–210.35 g at the time of dosing, were selected with 3 animals per group (Supplementary method 1 and Supplementary Table 1). The study was approved by the institutional IAEC (Approval No. IAEC/61/1185) and was conducted based on the requirements of the OECD Guideline for Testing of Chemicals, Test No. 423, “Acute Oral Toxicity- Acute Toxic Class Method”; adopted on December 17, 2001. As there was no toxicological information available about MAM Chooranam, the starting dose of 300 mg/kg body weight was selected for this study.

1. A dose of 300 mg/kg body weight was administered orally to Set I and animals were observed at 30 min, 1 hour, 2 hours, and 4 hours post-treatment. The treatment-related clinical signs and symptoms and mortality was observed in this SET I till scheduled treatment termination day 14.
2. A dose of 300 mg/kg body weight was administered orally to the Set II and animals were observed at 30 min, 1 hour, 2 hours, and 4 hours post-treatment. The treatment-related clinical signs and symptoms and mortality was observed in this SET II till scheduled treatment termination day 14.
3. A dose of 2000 mg/kg body weight was administered orally to Set III and animals were observed at 30 min, 1 hour, 2 hours, and 4 hours post-treatment. The treatment-related clinical signs and symptoms and mortality was observed in this SET III till scheduled treatment termination day 14.
4. A dose of 2000 mg/kg body weight was administered orally to the Set IV and animals were observed at 30 min, 1 hour, 2 hour, and 4 hour post-treatment. The treatment-related clinical signs and symptoms and mortality was observed in this SET IV till scheduled treatment termination day 14.

### 2.5 Cells and viruses

Vero E6 (African green monkey kidney epithelial) cells, Vero cells and RAW 264.7 (an immortalised murine macrophage cell line) cells were maintained in Dulbecco's Modified Eagle Medium **(**DMEM) supplemented with 10% inactivated fetal bovine serum **(**FBS) at 37 °C, 5% CO2 in an incubator. The concentration of FBS was reduced to 2% for the cytotoxicity and anti-viral assay. RAW 264.7 cells were used for accessing the antioxidant and anti-inflammatory potential whereas Vero E6 cells were used for cell viability assay and SARS-CoV-2 antiviral assays and Vero cells for CHIKV antiviral assay. For antiviral assays, Washington strain of SARS-CoV-2 (WA1/2020) was used at the MOI of 0.1 to infect Vero E6 cells. Viral amplification was performed in Vero E6 cells at 37 °C until the appearance of full cytopathic effects. Amplified virus was quantified using plaque assay. The ECSA strain of CHIKV isolate (CHIK/DEL/2010/01, Accession No. - JF950631.1) (27) was used for the present study which was propagated in C6/36 cells and used at 50 pfu/well of 96 well plate in Vero cells.

### 2.6 Cell viability assay

MTT assay was used to assess the cell viability of MAM extract using Vero E6 cells and RAW 264.7 cells and its ingredients using RAW 264.7 cells. For that, cells were seeded at the density of 10,000 cells per well in 96 well plate and incubated overnight. Next day, cells were rinsed with Phosphate buffered saline (PBS) and then treated with maximum non-toxic concentration (MNTD) of either MAM extract or with individual ingredient with subsequent 1:2 dilution/well in 2% MEM followed by incubation at 37 °C in a 5% CO2 incubator for 48 hours.

After the incubation, media was removed, cells were washed and 100 μl of freshly prepared MTT (0.5 mg/ml) solution was added to each well and plates were incubated for 4 hours at 37 °C in a 5% CO2 incubator followed by removal of supernatant and addition of DMSO (100 μl/well). Then, these plates were incubated at RT for 30 min to allow the dissolution of formazan complexes, the absorbance was recorded at 570 nm and cell survival was plotted with the help of GraphPad Prism 6.

### 2.7 Antiviral Assay

To access the antiviral activity of MAM extract, the following types of antiviral assays were performed using previously published protocols [31] with some modifications based on the virus tested. The CHIKV antiviral assay was used as per the published protocol using Vero cells. The following modifications were done in the protocol for antiviral assay for SARS-CoV-2.

Vero E6 cells were seeded at 10,000 cells per well in 96 well plate and incubated overnight. Next day, for pre-incubation assay, MAM extract was serially diluted (1:2), mixed with SARS-CoV-2 separately, incubated for 2 hours and then added to seeded Vero E6 cells, incubated for 2 hours again followed by replacement of media and subsequent incubation for 48 hours.

For co-incubation assay, Vero E6 cells were first incubated with SARS-CoV-2 for 2 hours, washed to remove unbound virus and incubated with serially diluted MAM extract in 10% DMEM for 48 hours. After 48 hours of incubation supernatant was collected for both the assays and then subjected to viral quantification using plaque assay.

### 2.8 Plaque Assay

A monolayer of Vero E6 (for SARS-COV-2)/Vero cells (for CHIKV) was formed by seeding 20,000 cells per well in 96 well plate and incubated overnight. Next day, media was aspirated and cells were washed with PBS. For SARS-CoV-2, 4 μl of collected sample was mixed with 96 μl media (total volume 100) and was added to first column already containing 100 μl media making the virus dilution to 1:50 followed by 1:2 serial dilutions (1:50 to 1:51200). Last column was used as negative control. Then these cells were incubated for 2 hours for viral adsorption followed by removal of media and addition of 2% CMC overlay medium. In case of CHIKV, first the antiviral assays were performed as described above which is immediately followed by addition of 2% CMC overlay medium. Then these plates were incubated either for 96 hours (SARS-CoV-2) or 72 hours (CHIKV). After the incubation, CMC overlay was removed and cells were fixed with 10 % formaldehyde for 2–3 hours. After the fixation, plate was rinsed with PBS and 0.25% crystal violet prepared in 30% methanol was added and incubated for 30 min at room temperature. Then, plate was washed and plaques were counted to calculate viral titre.

### 2.9 ABTS radical scavenging and SOD assay

The radical scavenging capacity of the extracts was evaluated by in vitro ABTS assays and total cellular SOD activity in cultured RAW264.7 cells. The ABTS^**+**^(2,2-azino-bis (3-ethylbenzthiazoline-6-sulfonic acid) radical scavenging assay was performed in 96-well microplates according to the manufacturer's instruction (Sigma, CS0790).

For SOD activity, cells were washed with ice cold PBS, lysed with lysis buffer and supernatants were assayed using a Sigma SOD assay kit (19160) using manufacturer's instruction. The SOD activity was measured at 450 nm using a microplate reader (Molecular Devices Spectramax M3, San Jose, CA, USA).

### 2.10 Intracellular Reactive Oxygen Species (ROS) generation

For intra-cellular ROS level was quantified by using 2,7-dichlorofluorescein diacetate (DCFH-DA) dye. RAW 264.7 cells were plated at a density of 0.5 × 10^5^ cells/mL in a 96 well plate, pre-treated with extracts for 4 hours and stimulated with 2 μg/ml of Lipopolysaccharide (LPS) for 24 hours. After incubation, cells were washed PBS and 10 μM DCFH-DA dye was added for another 30 min at 37 °C in dark. After incubation, fluorescence intensity was measured at 485 nm excitation and 535 emissions. The relative intensity with respect to LPS group represented here.

### 2.11 NO release assay and PGE2 production

The inhibitory effects of extracts on inflammatory mediator production were assessed for Nitrite release and PGE2 production. RAW 264.7 cells were seeded in 96-well plates at a density of 0.5 × 10^5^ cells per well. Cells were pre-treated with various concentrations of extracts for 4hrs followed by LPS stimulation for 24 h at 37 °C in an incubator with 5% CO_2_. After incubation cells supernatants were collected and nitrite level was analysed by the Griess reagent according to manufacturer's instructions (Himedia, CCK061). Similarly, PGE2 levels were assessed by using PGE2 ELISA kit (R&D Systems, KGE004B) in accordance with the instructions of the manufacturer. The standard curve was generated from solution provided in the kit and concentration of sample was analysed in accordance with the equation obtained through standard curve.

### 2.12 ELISA

The evaluation of pro-inflammatory cytokines present in supernatant collected from the treated cells was done by using ELISA (R&D Systems, KGE004B). The protocol was followed on the basis of manual provided in the kit. The level of cytokines was quantified in cell culture supernatant in accordance with the equation obtained through standard curve.

### 2.13 Statistical Analysis

Data presented here as mean ±standard deviation (SD). The statistical analysis of differences between the sample and LPS groups were analysed by one-way ANOVA. Significant differences were considered whose p value < 0.05.

## 3 Results

### 3.1 Characterization of MAM Granules

**Fig. 1:**
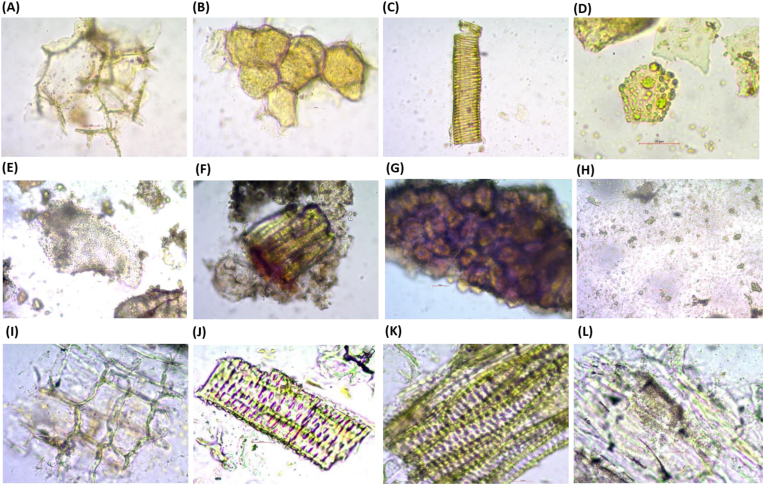
**Microscopy analysis of MAM powder.** (A–D) Parenchyma cells, Oleoresin cells, spiral vessels, and oil cells indicating *Curcuma longa*. (E–H) Perisperm cells, beaker-shaped stone cells, isodiametric stone cells, and starch grains indicating *Piper nigrum*. (I–L) Parenchyma cells, bordered pitted vessels, tracheid and starch grains indicating *Withania somnifera*.

**Fig. 2:**
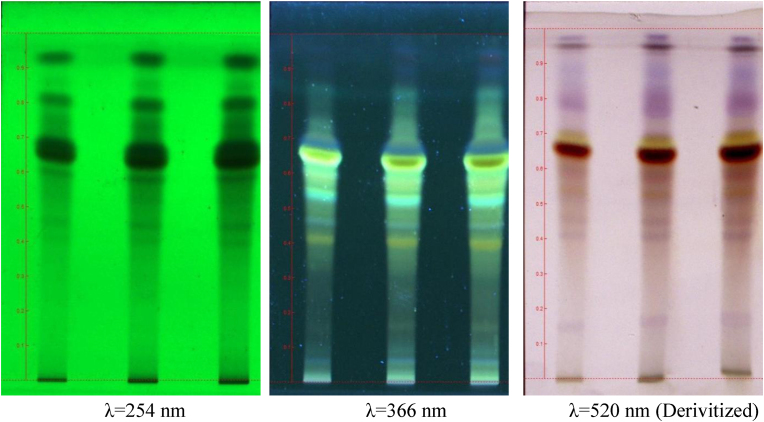
**TLC photodocumentation of MAM granules.** The TLC photos of MAM granules at UV 254, 366 nm and 520 nm after derivatization with vanillin-sulphuric acid showing presence of *Curcuma longa*, *Piper nigrum* and *Withania somnifera* respectively in the formulation.

The pharmacognostic study shows the authentication of herbal components used for the MAM Granule preparation. The special characteristics observed in the powder microscopy studies, confirm the presence of authenticated components. The presence of parenchyma cells, oleoresin cells, spiral vessels, and oil cells confirms the presence of the *Curcuma longa* in the formulation (Fig. 1, A-D). The presence of perisperm cells, beaker-shaped stone cells, isodiametric stone cells, and starch grains confirms the presence of *Piper nigrum* in the formulation (Fig. 1, E-H). The presence of parenchyma cells, bordered pitted vessels, tracheid and starch grains confirms the presence of *Withania somnifera* in the formulation (Fig. 1, I-L). The TLC photodocumentation is presented in Fig. 2.

The physico-chemical parameters showed 13.363 ± 0.62 % of loss due to drying at 105 °C which may be due to the moisture as well as the volatile oil content present in the ingredients of MAM granules. The total ash of 3.636 ± 0.020 % showed that the drug contains less inorganic content in which 2.380 ± 0.042 % were water soluble ash and nil foreign matter in the form of siliceous matter. The water-soluble extractive (14.27 ± 0.102 %) was higher than alcohol soluble extractive (6.49 ± 0.13 %) inferring the presence of high polar phytocompounds. The drug was slightly acidic with pH value of 6.0.

The colour and Rf of spots visualized under UV condition and after derivation with vanillin-sulphuric acid are presented in Table 1.

**Table 1:** R_f_ and Color of spots

^R^_f_	Colour	^R^_f_	Colour	^R^_f_	Colour
0.23	Green	0.06	Blue	0.16	Pale pink
0.28	Green	0.16	yellow	0.41	Pink
0.39	Green	0.40	Yellow	0.46	Pink
0.44	Green	0.45	Blue	0.51	Orange
0.52	Green	0.52	Blue	0.58	Orange
0.58	Green	0.66	Green	0.66	Brown
0.66	Green	0.68	Dark blue	0.70	Yellow
0.73	Green	0.76	Yellow	0.77	Violet
0.80	Green	0.83	Yellow	0.87	Violet
0.85	Green			0.94	Violet
0.92	Green				

### 3.2 HPTLC finger print profiling

Analysis of MAM reveals a great deal about their elemental composition. ICP-OES analysis of all the MAM revealed that Heavy metal like arsenic, lead, mercury and cadmium were not detected or within permissible limits.

**Fig. 3:**
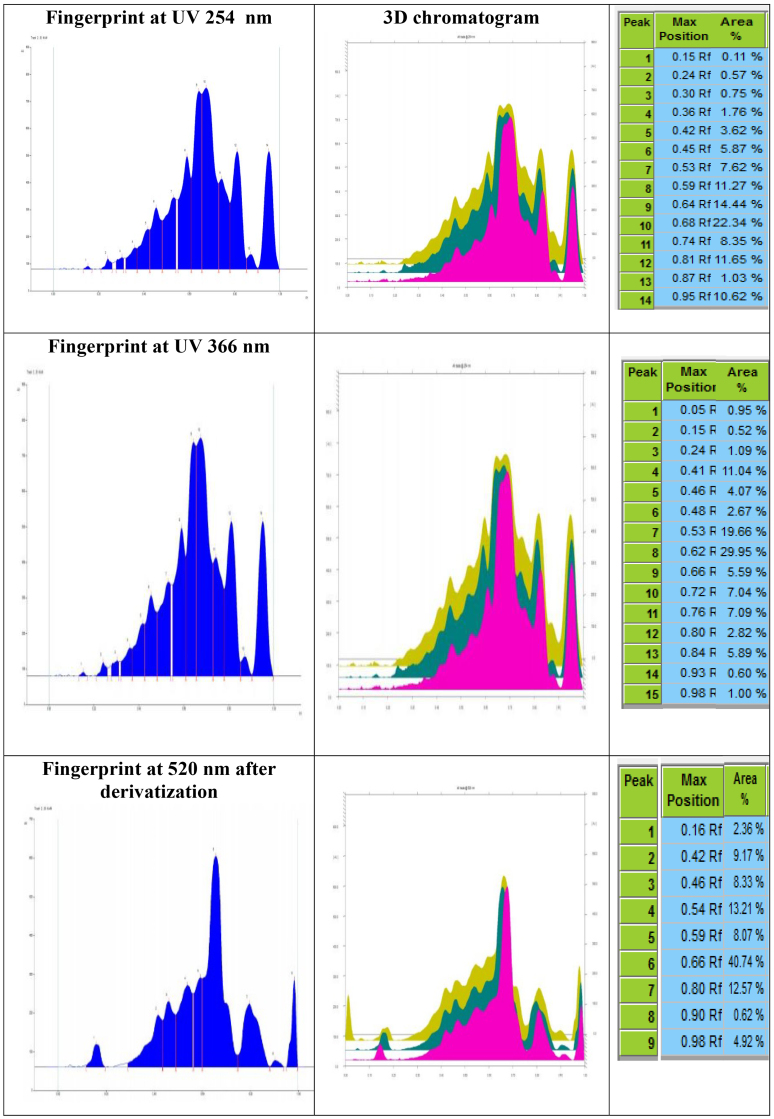
HPTLC Finger print profiling

The phytochemical screening of MAM Granules for the identification of phytochemical constituents by HPTLC Methods showed various peaks and Rf values as shown in Fig. 3.

### 3.3 MAM granules were well tolerated during *in-vivo* toxicity studies

MAM granules were evaluated for any toxicity in Wistar rats and no treatment-related clinical signs and symptoms were observed in all the animals till scheduled termination, ie, day 14. No mortality was observed at the dose level of 300 mg/kg b.wt. & 2000 mg/kg b.wt. during the experimental period. All the animals exhibited a progressive increase in body weight throughout the experiment period. Further, no external and internal abnormalities were observed during gross pathological evaluation at the end of day 14. At the dose level of 2000 mg/kg body weight, all the animals of set III (Animal no. 7, 8 & 9) and set IV (Animal no. 10, 11 & 12) were terminally sacrificed on day 14. No external and internal abnormalities were observed (Supplementary Tables 2–4).

Based on the above findings it was concluded the LD50 cut-off value of MAM granules in the acute oral toxicity study in Wistar rats was greater than 2000 mg/kg b.wt. As per Globally Harmonized System for the classification of chemicals (GHS) MAM Granules in Wistar Rats are classified in Category 5 or Unclassified for which Acute Toxicity Estimate (ATE) Value is 5000 mg/kg b.wt.

### 3.4 MAM extract potentially inhibits replication of RNA viruses (SARS-CoV-2 and CHIKV)

**Fig. 4:**
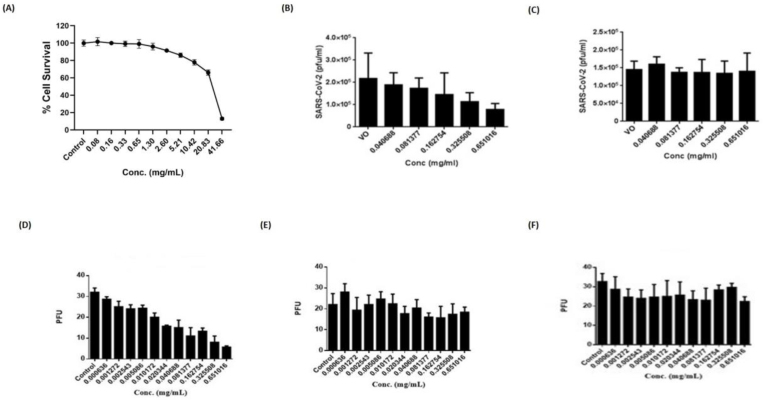
**Antiviral activity of MAM aqueous extract against SARS-CoV-2 and CHIKV**. (A) Shows cytotoxicity data of MAM aqueous extract treatment after 48 hours of incubation via MTT assay using Vero-E6 cells. Control represents untreated Vero-E6 cells. The bars represent mean ± SD. (B&C) demonstrates the antiviral activity of MAM aqueous extract during co-incubation and pre-incubation assay respectively with SARS-CoV-2 using Vero-E6 cells. VO represents untreated SARS-CoV-2 infected Vero-E6 cells and served as positive control to compare viral inhibition upon MAM treatment. The bars represent mean ± SD. (D–F) Shows antiviral activity for CHIKV using co-incubation, pre-treatment and post-treatment of MAM extract respectively using Vero cells. Control represents untreated CHIKV infected Vero cells. The bars represent mean ± SD.

As the first step, we analysed the cytotoxicity of the aqueous extract of MAM granules using an in vitro-based MTT assay. The MAM was showing 50% cell cytotoxicity (CC_50_) at the concentration of approximately 22 mg/ml at 48 hours of exposure. The maximum non-toxic dose (MNTD) was obtained around 0.65 mg/ml (Fig. 4A). Then, we assessed its capability to restrict virus infection. For this purpose, we chose two viruses, namely, SARS-CoV-2 and CHIKV, and systematically evaluated the ability of the extracts of the MAM nutraceutical to restrict virus growth. These assays were performed using maximum no-toxic dose (MNTD) and IC50 was calculated using MNTD as a starting concentration. We performed pre-incubation assay (SARS-CoV-2) and pre-treatment assay for CHIKV to investigate the protective effect against viral infections. Further, we employed co-treatment assay (CHIKV) and co-incubation (SARS-CoV-2) to study the possible inhibition of viral replication.

Our results investigating the antiviral activity of MAM showed inhibition in viral infectivity of ∼60% during co-incubation assay for both the viruses. We further determined the IC50 values i. e. ∼50 ug/ml for SARS-CoV-2 (Fig. 4B). For CHIKV, we also observed similar reduction in viral infectivity with an IC50 of ∼20.34 μg/ml (Fig. 4D).

Surprisingly, we could not observe any significant reduction in viral infectivity when MAM aqueous extract was pre-incubated with SARS-CoV-2 followed by infection of vero-E6 cells (Fig. 4C). Also, when we performed similar experiment as well as post-treatment assay with CHIKV, we observed no significant reduction again in viral infectivity (Fig. 4E&F).

### 3.5 MAM extract exhibit potent anti-oxidant and anti-inflammatory activities

**Fig. 5:**
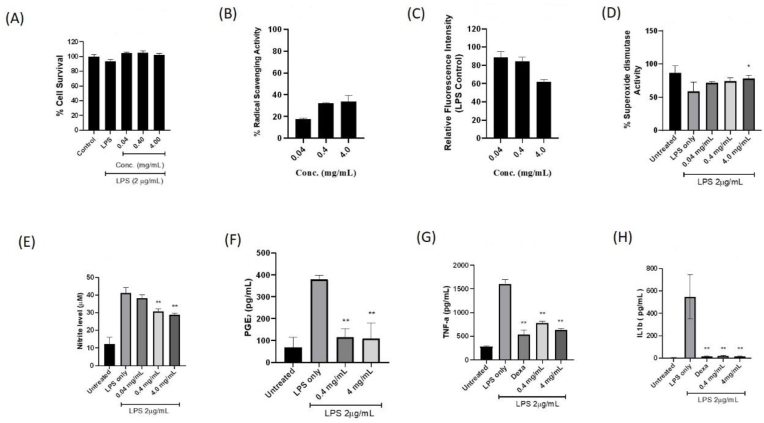
**Investigation of cytotoxicity, anti-oxidant and anti-inflammatory properties of MAM aqueous extract**. (A) Shows percentage cell survival of RAW 264.7 cells upon treatment of MAM via MTT assay. Control represents untreated RAW 264.7 cells. LPS represents RAW 264.7 cells treated with LPS. RAW 264.7 cells treated with 0.04, 0.4 and 4 mg/ml of MAM and 2 μg/ml of LPS showed no significant cytotoxicity. (B) demonstrate free radical scavenging capacity of MAM accessed via ABTS assay using RAW 264.7 cells. (C) Shows intracellular level of ROS induced by LPS and upon MAM treatment in RAW 264.7 cells. (D) Shows increase in intracellular SOD activity after MAM treatment in a dose dependant manner in RAW 264.7 cells. Untreated cells served as negative control and LPS treated cells as positive control. (E&F) shows effect on MAM treatment on the production of inflammatory mediators, NO measured as nitrite and PGE2 respectively using RAW 264.7 cells. Untreated cells served as negative control and LPS treated cells as positive control. (G&H) reflects the capability of MAM aqueous extract to counter the release of pro-inflammatory cytokines, TNF-alpha and IL-1 in RAW 264.7 cells. LPS treatment and untreated cells served as positive control and negative control respectively. The bars represent mean ± SD. One way ANOVA was used to calculate P-values. ∗P < 0.05, ∗∗P < 0.001 vs LPS treated Group.

In order to investigate the immune modulation properties of MAM aqueous extract, we evaluated the anti-oxidant and anti-inflammatory activity using RAW264.7 cells. As the first step, we accessed cytotoxicity of MAM extract on RAW264.7 cells and observed that the extract did not display significant cytotoxicity even at concentrations as high as 4 mg/ml (Fig. 5A). Based on these results, three concentrations, namely 0.04, 0.4 and 4.0 mg/ml, were used for all subsequent assays.

With respect to the scavenging activity, MAM extract demonstrated dose dependent decrease in the production of radical cation (ABTS·+) indicating significant radical scavenging capacity, ∼19%, 28% and 35% activity at 0.04, 0.4 and 4 mg/ml respectively (Fig. 5B). Afterwards, the intracellular level of ROS generated after the stimulation of RAW cells with Lipopolysaccharide (LPS) was measured which is well known to induce inflammatory response upon treatment. Only LPS treated cells were selected as positive control. We observed similar dose dependent reduction (16%, 20% and 40% reduction at 0.04, 0.4 and 4 mg/ml respectively) in the generation of intracellular ROS in cells treated with MAM extract suggesting a potent antioxidant activity (Fig. 5C).

Since SOD has been reported as the main enzyme participating in the first line defence as anti-oxidant against ROS [25], we further accessed the activity of intracellular SOD during the treatment of MAM. Monolayers of RAW264.7 cells were treated with the three concentrations of MAM extract. Untreated cells were used as negative control. LPS treatment was used to stimulate the generation of free radicals in these cells. Only LPS treated cells served as positive control. When we measured the SOD activity in MAM treated cells after LPS stimulation, the MAM treated RAW264.7 cells showed dose dependent increase (70%, 75% and 82% at 0.04, 0.4 and 4 mg/ml respectively) in SOD activity as compared to LPS treated positive control (Fig. 5D).

Taking all the above results together, such as the observed reduction in intracellular ROS and significant increase in the intracellular potent antioxidant SOD after in-vitro treatment with MAM extract, it clearly suggests that MAM extract have the capability to provide potent antioxidant activity.

After confirming the potent anti-oxidant activity of MAM extract, we subsequently investigated its anti-inflammatory property using the similar method. RAW 264.7 cells were either treated with LPS followed by MAM extract in dose dependent manner or left untreated (negative control). Cells treated with LPS only were used as positive control.

The production of nitric oxide (NO) and PGE2, two very important signalling molecules mediating the inflammatory response during viral infection and oxidative stress are reported to increase many folds, therefore we accessed the NO release and production of PGE2 after the MAM treatment and LPS stimulation. In our experiment, we indeed observed a significant reduction of production of these molecules in dose dependent manner in cells treated with MAM extract (Fig. 5 E & F).

Further, in order to investigate the possible mechanism of modulation of inflammatory response mediated by NO and PGE2, we analysed the effect of MAM extract on production of two key pro-inflammatory mediator cytokines, i. e. IL-1*β* and TNF*α*. The LPS stimulated cells had significantly increased the production of these cytokines as compared to their respective untreated controls. However, the treatment with MAM extract had suppressed the production of these cytokines in a dose-dependent manner (Fig. 5G &H). The inhibition of the proinflammatory cytokine activity of the highest concentration of extract (4 mg/ml) was noted to be working in similar manner with dexamethasone groups. The reduction of IL-1 beta by MAM extract was noted to exhibit the inhibitory activity with the dexamethasone group.

Collectively, our results demonstrate that MAM extract provide potent antioxidant and anti-inflammatory capability after treatment in RAW 264.7 cells.

### 3.6 The anti-oxidant and anti-inflammatory effects of MAM ingredients

**Fig. 6:**
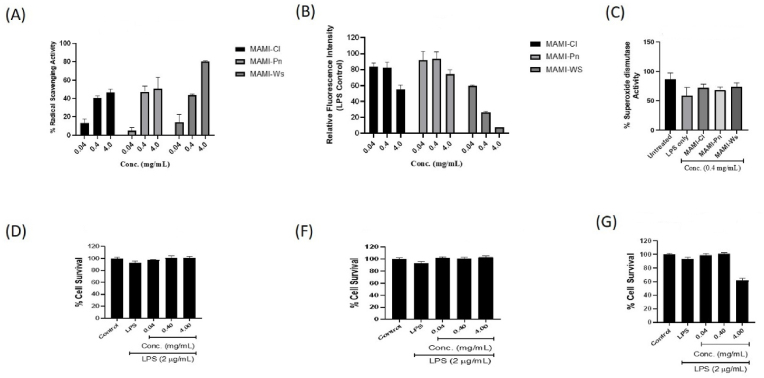
**Investigation of anti-oxidant activity and cytotoxicity of MAM ingredients** in RAW 264.7 cells. (A&B) demonstrates free radical and intracellular ROS scavenging capacity of each ingredient of MAM in a dose dependant manner assessed via ABTS and ROS assay respectively. Cl indicates *Curcuma longa*, Pn indicates *Piper nigrum* and Ws indicates *Withania somnifera*. (C) Shows increase in intracellular SOD activity after MAM treatment in RAW 264.7 cells. Untreated cells served as negative control and LPS treated cells as positive control. (D–G) Shows cytotoxicity of the three ingredients, Curcuma longa (Cl), Piper Nigrum (Pn) and *Withania somnifera* (Ws) respectively. The bars represent mean ± SD.

The potent antioxidant and anti-inflammatory properties displayed by MAM extract prompted us to examine the aqueous extracts of its ingredients, namely, *Curcuma longa*, *Withania somnifera* and *Piper nigrum*, in a similar manner to deduce the key ingredient that contributed to these aspects in the nutraceutical formulation. During our investigation of antioxidant potential of each MAM ingredient using above experimental set up, we observed that all three ingredients were capable of scavenging the generated free radicals and intracellular ROS in a dose dependent manner as showed in ABTS scavenging assay and ROS assay (Fig. 6A&B). All three ingredients also demonstrated increased intracellular SOD activity (Fig. 6C). This suggested that the observed potent antioxidant activity of MAM extract was due to the synergistic effect of these three ingredients. Amongst the three ingredients, *Withania somnifera* demonstrated highest antioxidant activity in all these assays (Fig. 6A–C). Moreover, in our assessment of NO release of all three ingredients, *Withania somnifera* showed highest inhibition in NO release indicating that the major immune modulation property is due to *Withania somnifera* in MAM extract (Fig. 6D). The investigation of cytotoxicity of these ingredients showed no significant cytotoxicity (Fig. 6E&F) with the exception of *Withania somnifera*, which showed ∼ 40% cytotoxicity at the concentration of 4 mg/ml (Fig. 6G).

## 4 Discussion

In the present study, we explored the Siddha medicinal system for developing a nutraceutical based therapeutic for COVID-19 and other viral infections. Several medicinal plants have been studied in this aspect and key phytochemicals, have been reported as active ingredients. In this study, we investigated one such Siddha nutraceutical viz. MAM granules that has been developed as per the basic principles of Siddha Gunapadam. The selection of these ingredients was done after studying the Siddha literature and a detailed analysis of the nutrient values of each ingredient revealed that these ingredients are rich in Vitamin A, C, E, zinc and manganese. Additionally, all these ingredients have been reported to have potent anti-oxidant and anti-inflammatory properties, when taken as diet supplement [[32], [33], [34]].

For instance, *Piper longum* L. Piperaceae, a fruit commonly known as Indian spice kali mirch, has been reported to show antiviral activity against Coxsackie virus type 3 (CVB3) due to the presence of α-pinene, β-pinene, limonene, myrcene, sabinene, camphene, α-thujone, piperitone, caryophyllene, *p*-cymene, α-terpinene, and piperamide [20]. Similarly, *Curcuma longa* L. (Zingiberaceae) is enriched with curcumenone, bisacumol, bisacurone, curcumenol, curcumadiol, and demethoxycurcumin. Curcumin inhibits SARS-CoV-2 replication in human cells, as previously reported for HIV-AIDS, herpes simplex virus (HSV) [20,21], chikungunya virus and Zika virus [35]. Likewise, Ashwagandha consists of sterols, alkaloids, saponins, amino acids and polysaccharides. Alkaloids such as ashwagandhine, cuscohygrine, trpine, isopelletierine, anaferine etc. have been isolated from Ashwagandha. The multiple numbers of sterols are isolated from this herb which includes withaferins, withasomidienone, withasomniferin A, withanolides, and withanone. Sitoindisides (VII, VIII, IX and X) and withaferin A. Withaferin A, a constituent of ashwagandha have a high binding affinity towards neuraminidase and inhibit neuraminidase of H1N1 influenza virus potently [17].

Various natural products and phytochemicals have been studied for their antiviral activity against SARS-CoV-2 [[36], [37], [38], [39], [40], [41]] and CHIKV [[42], [43], [44], [45]]. We also have studied siddha medicines and have reported to work as effective antivirals for RNA viruses in addition to possess the immune-regulatory properties [28,29]. Therefore, we also accessed the antiviral activity of MAM aqueous extract against two RNA viruses i. e. SARS-CoV-2 and CHIKV.

Similarly, many phytochemicals have been reported to inhibit viral replication [37,39,43], therefore, we investigated the ability of MAM to inhibit viral replication. We found significant reduction in viral replication of both RNA viruses with IC50 value of ∼50 μg/ml for SARS-CoV-2 and ∼20.34 μg/ml for CHIKV. This inhibition in viral replication indicated the possible interaction between MAM's phytochemicals and viral core proteins resulting in direct inhibition of SARS-CoV-2 and CHIKV viral core protein's function.

When a virus infects a cell, they initiate a cascade of cellular events that eventually leads to the propagation of the virus and death of the infected cell. The infected cell embarks on a series of cellular responses, first amongst them being oxidative stress. Increase in the production of reactive oxygen species and free radicals are the hallmark events of oxidative stress [46]. These free radicals tend to damage the cellular components and responsible for tissue injury [47]. Due to the compromised state of cellular homeostasis machinery and viral infection, cells were unable to scavenge these increased ROS and free radicals. One way to counter this oxidative damage is to provide molecules such as vitamin A, C, E, manganese, zinc, polyphenols and carotenoids as well as antioxidant enzymes (e. g. SOD) having capabilities to scavenge these generated free radicals and reactive oxygen species preventing the cellular and tissue damage. These antioxidants directly interact with free radicals, accept or donate electron/hydrogen atom and neutralize the unpaired condition or destroy them and become less reactive, long lived and least dangerous molecules as compare to the original free radicals [48,49]. This antioxidant activity can be supplemented both ways i. e. extracellularly such as dietary antioxidant and intracellularly (antioxidant enzymes).

During our exploration of the efficacy of MAM for its antioxidant property, we observed that the aqueous extract of MAM exhibited significant ability to scavenge the free radicals in commonly used radical cation-based antioxidant assay. The assay follows the principle of generation of cation free radicals (ABTS^•+^) via oxidation by metmyoglobin and H_2_O_2_. The oxidants work as reductant of radical ABTS^•+^ reducing the number of generated cation radicals [50]. This observed scavenging potential suggested that MAM could supply dietary antioxidants, thus significantly counter the sudden rise in generation of ROS and free radicals when contacted by virus.

As free radicals, ROS rise in virus infected cells, cellular homeostasis machinery employ intracellular antioxidants also in addition to the dietary antioxidants to reduce the generation of these reactive species for the prevention of cellular damages. Amongst them, SOD acts as first line of defence mechanism against these reactive species. The observed reduction in intracellular ROS generation and increase in intracellular SOD activity in our experiments in cells treated with MAM extract clearly suggested SOD as main intracellular defence mechanism against ROS and free radicals making MAM as suitable nutraceutical to manage oxidative stress and tissue damage during viral infection.

We further accessed the contribution of the three ingredients in anti-oxidant activity of MAM extract. During our assays, all three ingredients demonstrated radical scavenging capacity indicating the synergistic effect of observed activity of MAM extract. However, treatment with *Withania somnifera* showed potent decrease in production of intracellular ROS and increase in SOD activity as compared to other two ingredients. This indicates that the other two ingredients combine their activity synergistically with *Withania somnifera* thus providing potent anti-oxidant potential to MAM extract.

Viral infection mediated diseases often manifest as inflammation in target tissue leading to development of disease symptoms and sepsis shock in some cases. For instance, SARS-CoV-2 mediated COVID-19 disease leads to initiation of inflammatory response in lungs in response to viral infections. However, dysregulation of this response and subsequent cytokine production leads to development of ARDS [51]. Management of this dysregulated inflammatory response has been shown to be an effective strategy against COVID-19 and prevented significant morbidity and mortality. Similarly, CHIKV fever is characterized by severe joint inflammation and febrile illness and reduction in inflammation provides significant relief and effective management of disease symptoms [52]. These inflammatory responses can be activated by excess production of free radicals and ROS during oxidative stress [53]. Anti-inflammatory molecules suppress the uncontrolled inflammatory response and avoid these damages.

In view of these aspects, we evaluated the anti-inflammatory properties of MAM extract by accessing the production of two major inflammatory regulators i. e. NO and PGE2 in addition to measuring the production of two prominent pro-inflammatory cytokines, TNF-α and IL1-β, since both of these molecules serve as mediators of pro-inflammatory response against viral infections. We hypothesized that treatment of cells with MAM extract should reduce the production of these molecules in cells stimulated with LPS. Our data reveal that treatment with MAM extract effectively reduce the production of NO and PGE2. The decrease in inflammatory mediators was more prominent in PGE2 production as compared to NO release (Fig. 2). The excessive NO production under LPS stimulation [54] can be achieved via activation of promoter of the NOS2 gene by variety of transcriptional factors [55]. The observed reduction in excessive NO production by MAM treatment might be accomplished by one or combination of these activation mechanism, which require further investigation in detail. Similarly, PGE2 is synthesized primarily by cyclooxeganse-2 (COX-2) and can be induced by LPS treatment [55]. We observed prominent reduction in PGE2 production upon MAM treatment which could be attributed to the difference in the mechanism of production of these inflammatory mediators. NO production is achieved by different pathways hence inhibition of one or two pathways by MAM may not able to show potent inhibition in NO production whereas PGE2 production is done by COX2 only, hence its inhibition via MAM showed drastic decrease in its production. Moreover, potent reduction in the production of proinflammatory cytokines by MAM extract clearly suggest that MAM extract can be effectively used as anti-inflammatory agent for viral infections.

**6 Conclusion:** Therefore, with these data we propose that MAM could be safely used as antioxidant, anti-inflammatory and antiviral against management of disease mediated by other RNA viruses, not limited to SARS-CoV-2 and CHIKV. Moreover, the animal toxicity results support the designed formulation's safety and therapeutic effects and support its use as an alternative therapeutic supplement for COVID -19. Nevertheless, further studies are needed to verify the underlying mechanism of action of the MAM. The present study along with further preclinical studies and clinical trials can serve as a proof of concept of MAM to be a beneficial therapeutic supplement to combat viral infections.

## Sources of Funding

Central Council for Research in Siddha, Ministry of AYUSH, Govt. of India.

## Ethical approval

This study got approval from the Institutional ethical committee of Dabur Research Foundation, Ghaziabad. Uttar Pradesh and IAEC Approval No. is IAEC/611/185.

## Authors contribution

MR, VN – conceived the ideology of granules, involved in designing the protocols. DKV, MR, AH, GS, and SD involved in conception and design of experiments, acquisition of data, or analysis and interpretation of data. TKD – Involved in computational analysis and contributed in drafting the manuscript. SS supervised the overall experiments. DKV drafted the manuscript with inputs from MR, SD, SP, TKD and SS finalized the manuscript. SP, KK, SS – involved in final approval of the version to be published.

## Declaration of Generative AI in Scientific Writing

None

## Declaration of competing interest

The authors declare that they have no known competing financial interests or personal relationships that could have appeared to influence the work reported in this paper.
