# Supplementary material for: Evaluation of Withania somnifera based supplement for immunomodulatory and antiviral properties against viral infection
Source: J Ayurveda Integr Med. 2024 Oct 9;15(5):100955. doi: 10.1016/j.jaim.2024.100955 (PMC11693430; doi:10.1016/j.jaim.2024.100955)
Supplement: Multimedia component 1 [file mmc1.docx]

**Supplementary Methods 1:**

The animals were acclimatized for a period of 9, 11, 13, and 15 days for Set I, Set II, Set III, and Set IV respectively in the experimental animal room before the start of treatment. Post acclimatization, the animals were dosed with 300 mg/kg body weight (set I&II) and 2000 mg/kg body weight (set III&IV). For that, the indicated amount was triturated and final volume was made up to 10 mL using Milli Q water. The solution was freshly prepared and homogeneity was maintained during administration by vortex mixer. The animals were then observed once daily for any abnormalities for 14 days. The animals were fasted 15-18 hours before dosing and up to 3 - 4 h after dosing. Each animal was observed at regular intervals (at 30 minutes, 1, 2, and 4 h) on the day of dosing and twice daily up to fourteen days to assess general clinical signs of toxicity and for mortality/morbidity twice daily: once in the morning and once in the afternoon/evening throughout the observation period. The body weight of all the animals was also recorded on day 0 (before dosing), on day 7, and the day of termination day 14. Percentage change in body weight was calculated on day 14. After completion of the experiment, all the surviving animals were terminally sacrificed by carbon dioxide asphyxiation and gross pathological examination was carried out for external and internal examination.

**Supplementary Table 1: The detailed description of the animal groups.**

| Species | *Rattus norvegicus* |
| --- | --- |
| Strain | Wistar Rat |
| Source | GENTOX BIO SERVICES PVT. LTD., HYDERABAD  CPCSEA registration No.: 1242/PO/RcBiBt/S/08/CPCSEA |
| Sex | Female (nulliparous and non-pregnant) |
| Age | 11-13 weeks at the time of dosing |
| Body weight range | 160.91g – 210.35 g (at the time of dosing) |
| Number of Animals per group | 3 females per step |
| No. of groups | 4 |
| Acclimatization of animals | The animals were acclimatized for a period of 9, 11, 13 and 15 days for Set I, Set, Set III and Set IV respectively in the experimental animal room before start of treatment. The animals were observed once daily for any abnormalities. |
| Randomization | N/A |
| Identification of animals | During the acclimatization period the animals were marked with a temporary animal number using an indelible nontoxic marker pen on the tail after that animals were identified by permanent number by tail tattooing and by cage card labeling. The cage label contained the following details: Study No., Type of Study, Test Item Code, Animal Species/Strain, Dose, Group, Sex, and Cage No. / Animal No. and Sign & Date. |

**Supplementary Table 2: List of Clinical Signs.**

| **Clinical Signs** | **Set (Dose: mg/kg B. wt)** | | | |
| --- | --- | --- | --- | --- |
|  | **Set 1 (300)** | **Set II (300)** | **Set III (2000)** | **Set IV (2000)** |
| Normal | **+** | **+** | **+** | **+** |
| Found Dead | **-** | **-** | **-** | **-** |
| Abdominal Breathing | **-** | **-** | **-** | **-** |
| Abortion | **-** | **-** | **-** | **-** |
| Abrasion | **-** | **-** | **-** | **-** |
| Abscess | **-** | **-** | **-** | **-** |
| Aggression | **-** | **-** | **-** | **-** |
| Alopecia | **-** | **-** | **-** | **-** |
| Anorexia | **-** | **-** | **-** | **-** |
| Anuria | **-** | **-** | **-** | **-** |
| Apnea | **-** | **-** | **-** | **-** |
| Ascites | **-** | **-** | **-** | **-** |
| Ataxia | **-** | **-** | **-** | **-** |
| Blepharospasm | **-** | **-** | **-** | **-** |
| Catalepsy | **-** | **-** | **-** | **-** |
| Cataract | **-** | **-** | **-** | **-** |
| Chemosis | **-** | **-** | **-** | **-** |
| Chromodacryorrhea | **-** | **-** | **-** | **-** |
| Clonic Convulsions | **-** | **-** | **-** | **-** |
| Coma | **-** | **-** | **-** | **-** |
| Conjunctivitis | **-** | **-** | **-** | **-** |
| Convulsions  (Seizures) | **-** | **-** | **-** | **-** |
| Corneal Opacity | **-** | **-** | **-** | **-** |
| Cyanosis | **-** | **-** | **-** | **-** |
| Dermatitis | **-** | **-** | **-** | **-** |
| Diarrhea | **-** | **-** | **-** | **-** |
| Dorsal Recumbency | **-** | **-** | **-** | **-** |
| Dyspnoea | **-** | **-** | **-** | **-** |
| Edema | **-** | **-** | **-** | **-** |
| Emaciation | **-** | **-** | **-** | **-** |
| Emesis (Vomiting) | **-** | **-** | **-** | **-** |
| Epistaxis | **-** | **-** | **-** | **-** |
| Erythema | **-** | **-** | **-** | **-** |
| Erythematic eruption | **-** | **-** | **-** | **-** |
| Exophthalmos | **-** | **-** | **-** | **-** |
| Fasciculation | **-** | **-** | **-** | **-** |
| Fissuring | **-** | **-** | **-** | **-** |
| Flaccid Paralysis | **-** | **-** | **-** | **-** |
| Gasping | **-** | **-** | **-** | **-** |
| Haematuria | **-** | **-** | **-** | **-** |
| Haemorrhage | **-** | **-** | **-** | **-** |
| Hopping gait | **-** | **-** | **-** | **-** |
| Hyperactivity | **-** | **-** | **-** | **-** |
| Hyperaesthesia | **-** | **-** | **-** | **-** |
| Hyperthermia | **-** | **-** | **-** | **-** |
| Hypoaesthesia | **-** | **-** | **-** | **-** |
| Hypothermia | **-** | **-** | **-** | **-** |
| Iritis | **-** | **-** | **-** | **-** |
| Injury (wound) | **-** | **-** | **-** | **-** |
| Keratitis | **-** | **-** | **-** | **-** |
| Kyphosis | **-** | **-** | **-** | **-** |
| Lacrimation | **-** | **-** | **-** | **-** |
| Lateral Recumbency | **-** | **-** | **-** | **-** |
| Lethargy | **-** | **-** | **-** | **-** |
| Mass formation | **-** | **-** | **-** | **-** |
| Microphthalmos | **-** | **-** | **-** | **-** |
| Miosis | **-** | **-** | **-** | **-** |
| Moribundity | **-** | **-** | **-** | **-** |
| Mydriasis | **-** | **-** | **-** | **-** |
| Nostril discharges | **-** | **-** | **-** | **-** |
| Nystagmus | **-** | **-** | **-** | **-** |
| Obesity | **-** | **-** | **-** | **-** |
| Opisthotonos | **-** | **-** | **-** | **-** |
| Papule | **-** | **-** | **-** | **-** |
| Paralysis | **-** | **-** | **-** | **-** |
| Piloerection | **-** | **-** | **-** | **-** |
| Polyuria | **-** | **-** | **-** | **-** |
| Prolapsus | **-** | **-** | **-** | **-** |
| Prostration | **-** | **-** | **-** | **-** |
| Pruritus | **-** | **-** | **-** | **-** |
| Ptosis | **-** | **-** | **-** | **-** |
| Recumbence | **-** | **-** | **-** | **-** |
| Retinal vasculitis | **-** | **-** | **-** | **-** |
| Retropulsion | **-** | **-** | **-** | **-** |
| Rolling tail | **-** | **-** | **-** | **-** |
| Rolling gait | **-** | **-** | **-** | **-** |
| Salivation | **-** | **-** | **-** | **-** |
| Scale | **-** | **-** | **-** | **-** |
| Sloughing | **-** | **-** | **-** | **-** |
| Snuffle | **-** | **-** | **-** | **-** |
| Somnolence | **-** | **-** | **-** | **-** |
| Spastic locomotion | **-** | **-** | **-** | **-** |
| Spastic paralysis | **-** | **-** | **-** | **-** |
| Sternal Recumbency | **-** | **-** | **-** | **-** |
| Sweating | **-** | **-** | **-** | **-** |
| Swelling | **-** | **-** | **-** | **-** |
| Tachypnea | **-** | **-** | **-** | **-** |
| Tail erection (Straub tail) | **-** | **-** | **-** | **-** |
| Tonic convulsion | **-** | **-** | **-** | **-** |
| Tonic- Clonic  convulsion | **-** | **-** | **-** | **-** |
| Tremors | **-** | **-** | **-** | **-** |
| Unusual locomotion | **-** | **-** | **-** | **-** |
| Vasodilation | **-** | **-** | **-** | **-** |
| Vesicle | **-** | **-** | **-** | **-** |
| Vocalization | **-** | **-** | **-** | **-** |
| Waddling gait | **-** | **-** | **-** | **-** |
| Writhing | **-** | **-** | **-** | **-** |
| Wry Neck (Torticollis) | **-** | **-** | **-** | **-** |

Keys: + = Present, - = Absent

**Supplementary Table 3: Summary of Mean Body Weight (g).**

| **Group** | **Dose (mg/kg b. wt.)** | **Day** | **No. of Animals** | **Mean** | **SD** |
| --- | --- | --- | --- | --- | --- |
| **Set I** | **300** | 0 | 3 | 179.21 | 14.73 |
|  |  | 7 | 3 | 191.52 | 16.00 |
|  |  | 14 | 3 | 202.85 | 16.72 |
| **Set II** | **300** | 0 | 3 | 181.48 | 19.64 |
|  |  | 7 | 3 | 194.93 | 21.33 |
|  |  | 14 | 3 | 206.45 | 15.93 |
| **Set III** | **2000** | 0 | 3 | 185.71 | 13.35 |
|  |  | 7 | 3 | 199.93 | 13.48 |
|  |  | 14 | 3 | 213.76 | 13.54 |
| **Set IV** | **2000** | 0 | 3 | 200.34 | 13.07 |
|  |  | 7 | 3 | 212.02 | 12.85 |
|  |  | 14 | 3 | 220.15 | 8.53 |

**Supplementary Table 4: Summary of Gross Pathological Findings of Vital Organ.**

| **Organs** | **Set (Dose: mg/kg B. wt)** | | | |
| --- | --- | --- | --- | --- |
|  | **Set 1 (300)** | **Set II (300)** | **Set III (2000)** | **Set IV (2000)** |
| Brain | NAD | NAD | NAD | NAD |
| Heart | NAD | NAD | NAD | NAD |
| Liver | NAD | NAD | NAD | NAD |
| Spleen | NAD | NAD | NAD | NAD |
| Kidney | NAD | NAD | NAD | NAD |
| Ovary | NAD | NAD | NAD | NAD |
| Uterus | NAD | NAD | NAD | NAD |

Keys: NAD = No Abnormality Detected.
